# Beyond protist generalizations: different evolutionary trajectories in closely related microbial species

**DOI:** 10.1093/ismeco/ycag218

**Published:** 2026-03-08

**Authors:** Iva Jadrná, Zuzana Škvorová, Pavel Škaloud

**Affiliations:** Department of Botany, Faculty of Science, Charles University, Benátská 2, 128 00 Praha 2, Czech Republic; Department of Zoology, Faculty of Science, Charles University, Viničná 7, 128 00 Praha 2, Czech Republic; Department of Botany, Faculty of Science, Charles University, Benátská 2, 128 00 Praha 2, Czech Republic; Department of Botany, Faculty of Science, Charles University, Benátská 2, 128 00 Praha 2, Czech Republic

**Keywords:** biogeography, dispersal, ecology, population differentiation, protists, restriction-site-associated DNA (RAD) sequencing, speciation, *Synura*

## Abstract

The processes underlying microbial diversity are inherently complex, so it is highly challenging to establish universal explanations and theoretical models of speciation. Using a population genetic approach, our study reveals that even closely related protist taxa with similar distributions, inhabiting overlapping ecological niches and comparable abundances, employ distinct speciation strategies. We investigated the population structure of two related species, *Synura petersenii* and *Synura glabra* (Chrysophyceae, Stramenopiles), across Europe. Using single-digest restriction-site-associated sequencing, we analyzed genomes of both species from a total of 43 distinct water bodies. A population pattern was present in both algal species, demonstrating their recent divergence and ongoing speciation. Nevertheless, the underlying drivers of genetic differentiation varied substantially. *Synura petersenii* exhibited a population pattern primarily associated with environmental factors, with conductivity emerging as the strongest habitat predictor of genetic structuring. In contrast, *S. glabra* demonstrated strict geographical boundaries and formed populations primarily according to geographical distance. We propose that divergences in population patterns between the two studied species arise due to differing mechanisms of distribution. By employing a balanced sampling design with comparable geographic coverage for both species, our study enables a robust comparison of their divergence and provides new insights into the complexity of speciation.

**Graphical Abstract:**
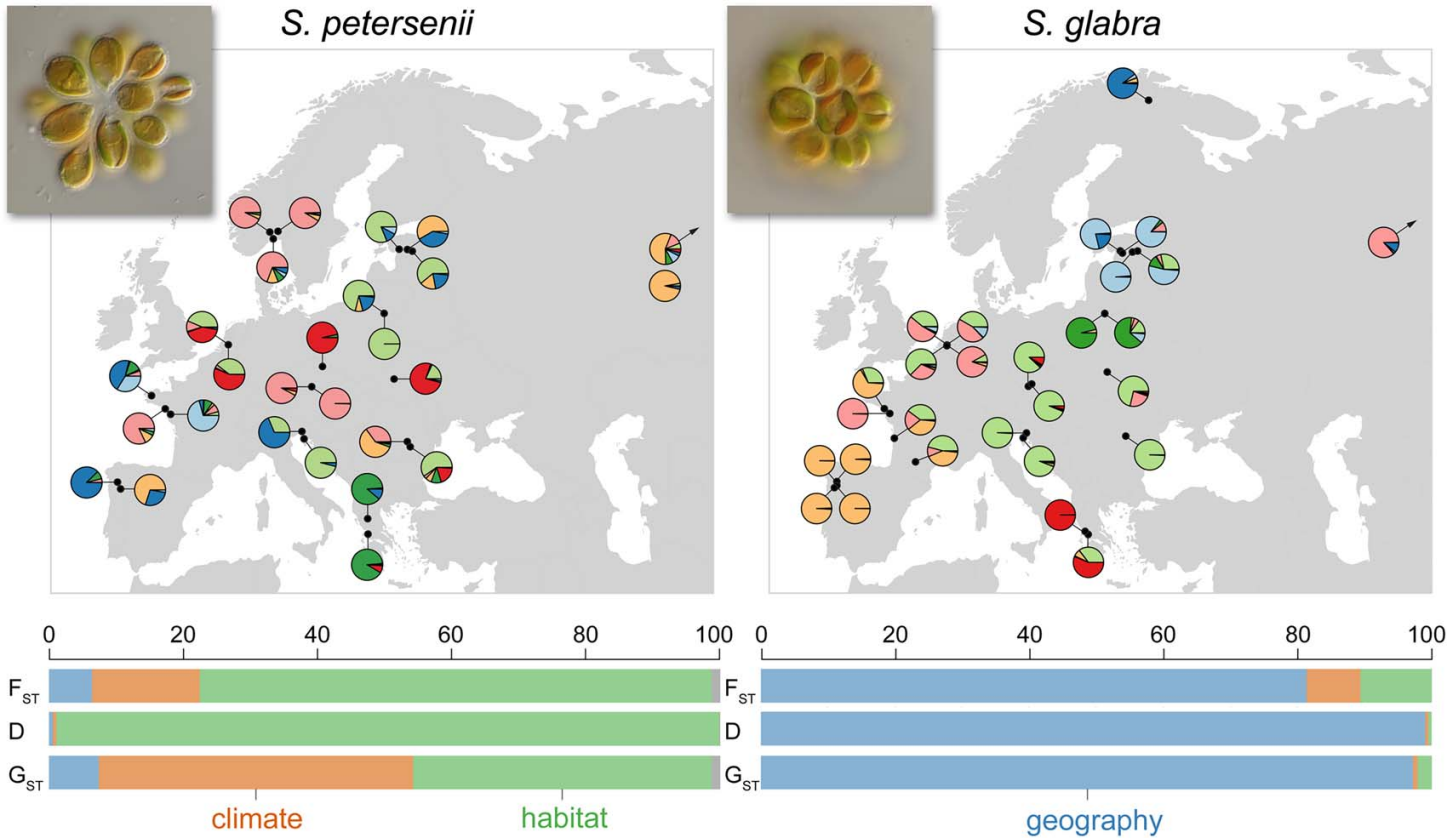

## Introduction

Adaptation to one’s environment promotes growth and success. When species expand to new locations, they encounter novel conditions that may require adaptation often accompanied by genetic differentiation [1–3]. Deeper insights into the complex phenomenon of species diversification enable recent advances in genome analyses, including high-throughput sequencing technologies ranging from whole-genome to environmental sequencing, demonstrating that the underlying principles of speciation are applicable across all eukaryotes, including microscopic taxa such as protists [4]. Despite their small size and common ability to produce persistent stages that could facilitate unrestricted dispersal [5], speciation models and differentiation drivers described for macroorganisms are increasingly recognized in protists as well [6–8]. Natural barriers such as sea currents can prevent the arrival of new protist migrants and thereby significantly restrict gene flow and contribute to the development of genetically differentiated lineages [9, 10]. This process, known as allopatric speciation, is often discussed in protists in the context of distance decay rather than the presence of discrete geographic barriers [11–13]. However, it is important to distinguish between vicariance and founder-event speciation, two mechanisms of allopatric diversification. Vicariance refers to speciation resulting from the fragmentation of a once-continuous population by the emergence of a geographic or ecological barrier (e.g. mountain formation, continental drift, habitat fragmentation, or hydrological isolation), whereas founder-event speciation occurs when a small group disperses to a new, isolated location (such as an island), leading to rapid divergence due to genetic drift and limited gene flow [11, 13]. Hydrologically isolated lakes provide one example of such isolation, as they impose distinct ecological pressures and may function as ecological islands for limnetic protists [14]. In contrast, sympatric speciation driven by ecological or temporal segregation has also been widely reported [8, 15–17]. These processes can result in detectable differences in community composition or population genetic structure even across relatively small geographic distances [18, 19].

Despite these insights, many aspects of protist speciation remain unresolved. In particular, the relative roles of geographic distance, historical events, and environmental gradients in shaping species abundance, distribution, population differentiation, and ultimately speciation are still poorly understood. Addressing these questions is essential for understanding the evolutionary processes that generate protist diversity and requires geographically extensive, environmentally diverse, and taxonomically well-resolved datasets. Each study yields different results depending on the species, habitat, study area size, and methodology used. The geographical scale at which individuals form a single population varies greatly. In some cases, population genetic structure is detectable at very fine spatial scales [20], whereas in others such differentiation is detectable across large geographic distances [21, 22]. Such variability suggests that each case may be unique. To what extent are modes of speciation and the factors that drive them comparable within lineages or genera? Could phylogenetically closely related protist species exhibit similar patterns of differentiation? The majority of studies focus on speciation drivers of single species or attempt to delineate species within a single genus. Comparative analyses are often obscured by inconsistent sampling, divergent interpretations, or unclear species boundaries.

To address these questions, we focused on two closely related freshwater protist species with cosmopolitan distribution: *Synura petersenii* and *Synura glabra* (Chrysophyceae, Stramenopiles). Both are colonial golden algae composed of heterokont flagellates that form spherical, rotating colonies in the water column [23]. They belong to a lineage that underwent rapid diversification during the Late Miocene, ~14 million years ago, resulting in the emergence of numerous closely related species [24, 25]. Although morphologically similar under light microscopy, the two species can be distinguished using transmission electron microscopy by the structure of their siliceous scales. *Synura glabra* was initially considered only as a form of *S. petersenii* due to their comparable scale ultrastructure in silica-depleted water [26], but later findings, including molecular analyses, confirmed the status of *S. glabra* as a separate species [27]. They represent the two most common *Synura* species in Europe and have similar but not identical ecological requirements, and thus often occur in the same localities [28]. Worldwide molecular investigations have demonstrated that the genus *Synura* encompasses both cosmopolitan and endemic cryptic species, which often co-occur in some localities [29]. Partly, the cryptic diversity of *Synura* was puzzled out by the integration of genetic and morphological approach and proven in numerous studies [27, 30, 31], but the population patterns and genetic differentiation remained unclear. The only study on population differentiation in the genus *Synura* demonstrated that the populations of *S. petersenii* are shaped by environmental conditions combined with the effects of long-distance decay [8].

Moreover, unlike many protists that remain uncultured, and are therefore less accessible to genome-wide approaches, investigated *Synura* species can be maintained under laboratory conditions, enabling biomass-dependent genomic analyses. Consequently, other protists are often studied using single-cell or community-based approaches [32–34] that provide more limited resolution for population-level inference. These characteristics make *Synura* particularly well suited for high-resolution studies of population genetic structure, differentiation, and speciation in freshwater protists.

Building on previous work [8], this study aimed to provide a more comprehensive assessment of the population genetic structure of *S. petersenii* by expanding the geographic scope of the study and substantially increasing the sampling effort. We further aimed to compare the population genetic structure of *S. petersenii* and its closely related sister species, *S. glabra*, across diverse environments and broad geographic distances. Using single-digest restriction-site associated DNA (sdRAD) sequencing to generate genome-wide single-nucleotide polymorphism (SNP) data, we investigated the factors shaping genetic differentiation in both species. Moreover, we evaluated how differences in their life-history strategies may contribute to their contrasting population genetic patterns. Through this comparative approach, we sought to gain insights into the evolutionary processes underlying population divergence and speciation, thereby contributing to a broader understanding of protist evolution.

## Materials and methods

### Sampling, isolation, and genotyping of cultures

Samples were collected from standing water bodies in 12 geographically distant regions across Europe, along with one remote site in continental Asia (Fig. S1), covering a wide range of habitats and environmental conditions. pH and conductivity of sampled water bodies were measured by WTW340i (WTW GmbH, Weilheim, Germany). On the day of sampling, monoclonal cultures of *Synura* species identified as belonging to section *Petersenianae* by light microscopy were established by micropipetting into 96-well plates containing WC liquid medium [35]. After 2 weeks of cultivation at 14°C under constant illumination of 40 mmol photons m^−2^ s^−1^, 60 μl of culture was harvested for genotyping. The internal transcribed spacer (ITS) regions of the ribosomal DNA genes were sequenced using primers Kn1.1 [36], Chryso_ITS_R [25], and ITS4 [37], following [28]. PCR products were purified using ExoSAP-IT™ (Applied Biosystems) and sequenced in Macrogen Europe (Amsterdam, Netherlands). Sequence alignments were manually built and curated in MEGA6 [38]. Strains were assigned to *S. petersenii* or *S. glabra* based on ITS rDNA sequences that matched the known intraspecific variability of each species as documented in our previous pan-European survey [28] and therefore did not require complete identity with reference sequences. Only individuals identified as these two species, both of which exhibit consistently high culturing success, were retained. To assess the population structure, we have randomly selected up to five cultures with confirmed molecular identification from each locality. The final dataset consisted of 126 cultures of *S. petersenii* representing 27 localities and 93 cultures of *S. glabra* representing 28 localities, ensuring balanced geographic representation for both species (Table S1).

### Single-digest RAD-seq library preparations

We used a sdRAD library protocol modified from [39, 40]. All protocol modifications are described in detail in [8]. Briefly, the DNA from 150 ml of well-grown monoclonal cultures was isolated using the DNeasy Blood and Tissue Kit (Qiagen, Hilden, DE, USA). The obtained DNA concentration was measured using Qubit dsDNA High Sensitivity Assay Kits (Thermo Fisher Scientific, MA, USA). Then, 1 μg of genomic DNA was digested with the restriction enzyme *Sbf*I-HF (New England Biolabs, MA, USA). The P1 adapters with unique 7-bp and 10-bp barcodes were ligated using T4 DNA ligase (200 000 U/ml; New England Biolabs) to the restricted samples overnight at 22°C. The samples with corresponding P2 barcodes were mixed, purified using the MinElute PCR Purification Kit (Qiagen), and shortly sonicated using the M220 Focused-Ultrasonicator (Covaris, MA, USA) targeting fragments of size ~500 bp. Afterwards, the libraries were prepared for ligation of the P2 adapters with 6-bp barcodes. 5′ or 3′ overhangs were converted using the Quick Blunting Kit protocol (New England Biolabs), directly followed by adding a polyA tail at the 3′ ends with Klenow Fragment (5 U/ml; New England Biolabs). The libraries were purified with the MinElute PCR Purification Kit (Qiagen), and P2 adapters were attached using T4 DNA ligase (200 000 U/ml; New England Biolabs) overnight at 22°C. After re-purification (MinElute PCR Purification Kit; Qiagen), the final amplification was performed with Q5 High-Fidelity 2X Master Mix (New England Biolabs) and 10 μM Solexa primers (forward primer P1: 5′-AAT GAT ACG GCG ACC ACC GA-3′; reverse primer P2: 5′-CAA GCA GAA GAC GGC ATA CGA-3′). The DNA fragments of 250–600 bp in length were selected by Pippin Prep (Sage Science, MA, USA). The concentration of the final libraries was checked using Qubit dsDNA High Sensitivity Assay Kits (Thermo Fisher Scientific) and pooled equally. Sequencing was carried out using Illumina HiSeq2500 with TruSeq v4 chemistry (Macrogen, Korea) with 20% PhiX control.

### RAD-seq data assembly

Due to the phylogenetic distance between *S. glabra* and *S. petersenii*, RAD-seq libraries were analyzed separately in Stacks2 [41]. The pipeline *process_radtags* was used to demultiplex the individuals and to remove low-quality data and sequences without the restriction site of the enzyme *Sbf*I. A *de novo* assembly was built using *denovo_map.pl* pipeline with selected optimal parameter combinations: m = 5 (minimum number of reads to seed a stack), M = 3 (maximum distance allowed between stacks), and n = 3 (number of mismatches allowed between sample loci). PCR duplicates based on insert length were removed. Another filter program Kraken2 [42] excluded culture contaminants, such as bacteria and the heterotrophic protist *Bodo*. Several parameters for loci filtering were tested using different R thresholds in stacks populations (minimum percentage of individuals across populations required to process a locus; values of 3, 5, 10, and 20 were evaluated). For each dataset, maximum-likelihood phylogenetic trees were inferred in IQ-TREE [43], using ultrafast bootstrapping with 2000 replications and GTR + I + G substitution model. After assessing tree topology and branch support, the optimal strategy was identified as R = 10 (Fig. S2). Only the first SNP per locus was selected to avoid linkage. Datasets needed for downstream analyses in STRUCTURE were exported directly in Stacks2 [41] into genepop and vcf formats or converted with PGDSpider [44]. Data processing was conducted using R v. 4.0.2 [45]. The package vcfR 1.16 [46] was used to estimate both the proportion of missing data and mean read depth.

### Population structure analyses

Population structure of *S. petersenii* and *S. glabra* was calculated from the filtered dataset of unlinked SNPs using a Bayesian cluster analysis implemented in the software STRUCTURE [47]. The analyses were run using ParallelStructure [48] on MetaCentrum computing resources operated by CESNET with *K* = 1–10, 50 000 burn-in steps, and 100 000 MCMC generations after burn-in, running 50 iterations for each *K*. The server CLUMPAK [49] was used to visualize summarized results, employing the CLUMPP LargeKGreedy algorithm. These results were mapped by geographical origin in R. The most probable *K* was estimated with StructureSelector [50], following [51]. Principal component analysis (PCA) and principal coordinates analysis (PCoA) were constructed to illustrate the population structure using the packages adegenet and ade4 [52] implemented in R. PCoA was based on Nei’s distances converted to Euclidean using the package StAMPP [53] implemented in R.

### Association between environmental predictors and genetic data

To determine whether genetic diversity was associated with known environmental variables, we tested correlations between the final filtered datasets of SNPs and selected climatic, habitat, and geographical variables. Climatic data were obtained from CHELSA v2.1 Climatologies database [54] with long-term mean values for the period 1981–2010. Habitat information was measured at sampling sites (pH values and conductivity/ionic content) or derived from the SoilGrids 2.0 database [55]. Spatial distances based on the geographic coordinates were transformed to principal coordinates of neighbor matrices in R, using the packages BoSSA and vegan [56]. All variables were standardized, and a set of uncorrelated environmental variables was selected. Uncorrelated variables were identified by calculating Pearson correlation matrices for all climatic and habitat variables across localities and selecting only those that formed weakly correlated clusters in hierarchical correlation plots (corrplot). Ten climatic variables were chosen: annual mean temperature (bio1); isothermality (bio3, ratio of diurnal variation to annual variation in temperatures); mean daily mean air temperatures of the wettest quarter (bio8); annual precipitation amount (bio12); precipitation amount of the driest month (bio14); precipitation seasonality (bio15); net primary productivity (npp); mean monthly near-surface wind speed (sfc); climate moisture index (cmi, average monthly climate moisture index over 1 year); amount of liquid water if snow is melted (swe). Seven habitat variables were selected: soil sand content (sand); soil clay content (clay); soil silt content (silt); soil organic carbon content (occ); soil pH; pH and conductivity of water body measured during sampling. Latitude and longitude were included as geographical variables.

Genetic-environmental associations at the individual level were tested using variation partitioning via the varpart function in the vegan package [57] in R, followed by ANOVA to test for significance. Predictors included filtered SNPs and 10 climatic, 7 habitat, and 2 geographic variables. Moreover, to detect the associations of environmental variables with genetic diversity, we performed a redundancy analysis (RDA), which allows both the predictor and the response variables to be multivariable. Since missing data were not allowed, they were replaced by the most common genotype. To verify that imputing these values did not bias the results, we repeated the analyses using alternative SNP filtering thresholds that differed substantially in the proportion of missing data. All datasets produced highly similar PCA and RDA patterns, indicating that the genetic-environmental associations were robust to different levels of missing data.

The relative contributions of climatic, habitat, and geographical predictors were estimated using generalized linear mixed modeling (GLMM) in R v. 4.1.0, with the package MCMCglmm [58]. *D*_Jost_, *G*_ST,_ and *F*_ST_ genetic pairwise distances were used as response variables. *F*_ST_ was determined in STACKS and the others were computed in R with the package FinePop [59]. Climatic and habitat predictors were derived from PCA-based Euclidean distances, while geographical predictors used spatial distances between populations. Analyses accounted for locality-pair independence with pairwise matrices, and model comparison was based on deviance information criteria (DIC).

Finally, we employed the gradient forest analysis in R 3.6.3, using the gradientForest package 0.1-18 [60], to evaluate which environmental factors most effectively explained observed genetic variability. The first 10 PCA axes of genetic diversity from the final dataset of SNPs were used as response variables. To take the hierarchical sampling design into account, the locality was set as a random factor. In total, 200 separate analyses were conducted based on 26/27 randomly selected samples (one per each locality), and the models were combined using the “combinedGradientForest” function. Gradient models were fitted with 2500 trees, the correlation threshold set to 0.5 and maxLevel = 3.

## Results

### RAD-seq data recovery

The quality-filtered RAD-seq dataset included 9653 SNPs with 75.7% and 8169 SNPs with 74.9% of missing data for *S. petersenii* and *S. glabra*, respectively. The average coverage for loci in the datasets was 36.6× for *S. petersenii* and 37.2× for *S. glabra*, with minimum coverage values of 4.3× and 2.0×, respectively, indicating that all retained SNPs exceeded the depth required for reliable genotype calling. Considering that, only loci with a small proportion of missing data were left which ensured a quality informative value of obtained datasets. The recovery for *S. petersenii* loci used in the final dataset was 851 SNPs (41.5% of missing data) and 849 SNPs remained for *S. glabra* (32.8% of missing data). The summary of population genetic metrics is given in Table S2.

### Population genetic structure

**Figure 1:**
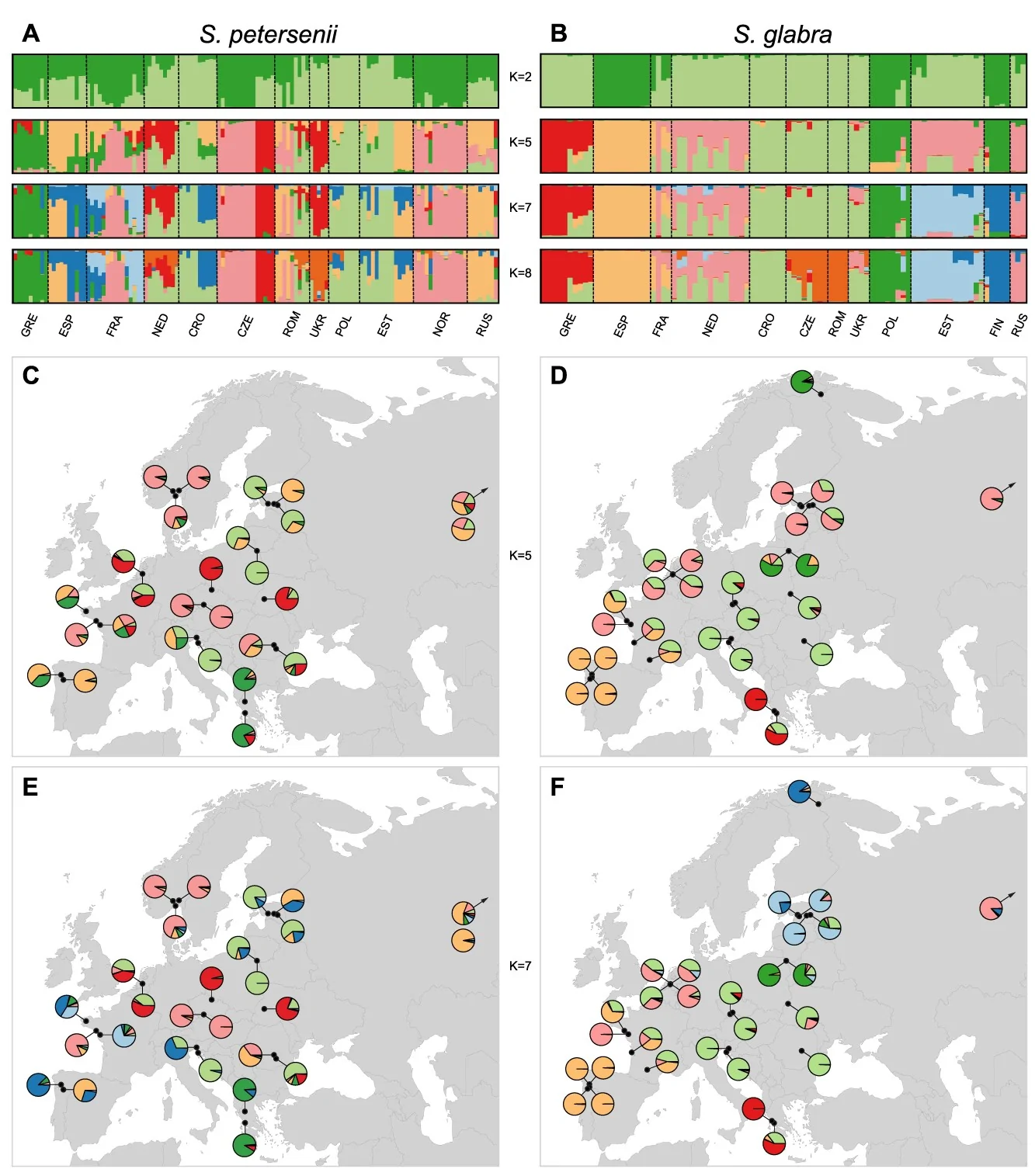
Population structure of *S. petersenii* (A, C, E) and *S. glabra* (B, D, F). (A, B) Structure plots for clustering values *K* 2, 5, 7, and 8. Each vertical bar corresponds to a strain, with the distinct segments within each bar indicating the assignment probabilities to each genetic cluster (K). Bottom labels indicate country abbreviations. (C–F) Geographic distribution of clusters for *K* 5 and *K* 7.

Both species showed high intraspecific genetic divergence. CLUMPAK identified two populations for *S. petersenii* and eight populations for *S. glabra* as the most likely clustering solutions based on the Δ*K* criterion. However, the Δ*K* peaks were only weakly pronounced, indicating limited statistical support for a single optimal number of clusters. In STRUCTURE analyses, such patterns typically arise when population structure is gradual or hierarchical rather than clearly partitioned into discrete genetic groups. Therefore, alternative clustering solutions may be equally plausible. To aid interpretation, we visually inspected the assignment plots across a range of *K* values. This approach is commonly used when Δ*K* does not provide a strong signal because gradual or weak structure often manifests as mixed ancestry proportions across individuals. In our case, solutions with five to seven clusters provided a more informative and biologically meaningful representation of the underlying genetic structure in both species (Fig. 1A and B).

Clustering of *S. petersenii* into two genetic populations revealed one group from Norway, Czechia, France, and Greece, while the second one was located on the territory of Croatia, Estonia, the Netherlands, Poland, and Ukraine (Fig. 1A). However, even when the number of inferred clusters was increased, individuals from most localities still showed mixed cluster assignment, suggesting only weak genetic differentiation and possible gene flow among regions. More refined clustering into five or seven groups revealed clearer, though still partially overlapping, geographic patterns (Fig. 1C and E). Samples from Greece consistently formed a distinct population regardless of the number of clusters. Interestingly, some individuals from Croatia grouped together with samples from Estonia and Poland, indicating complex patterns that do not strictly follow the geographic proximity.

A different pattern of genetic structure was observed for *S. glabra*. Clustering into two populations separated samples from Finland, Poland, and Spain, with evidence of gene flow from France to the rest of Europe (Fig. 1B). Increasing the number of populations made individuals from these countries fully distinct from others, indicating strong geographical structuring. With five or seven populations, samples separated into groups including Spain, Greece, Finland, Estonia, Poland, and eastern Europe, while coastal regions of the Netherlands and France showed distinct admixture signals (Fig. 1D and F). Notably, samples from a remote Asian locality were consistently grouped with European populations, even at higher numbers of clusters.

**Figure 2:**
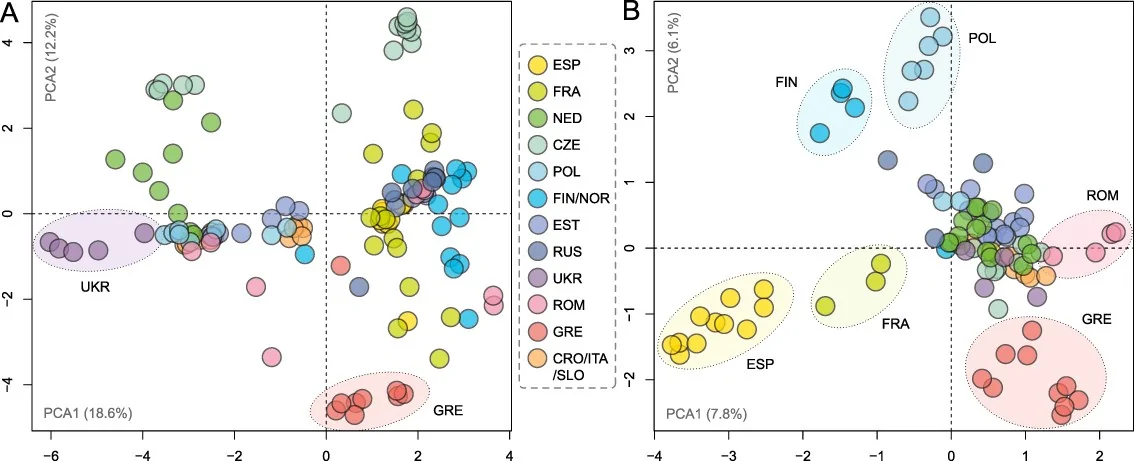
PCA ordination plots showing genetic relationships between the strains of *S. petersenii* (A) and *S. glabra* (B) based on SNPs acquired from sdRAD-seq. Each point represents an individual strain, grouped according to its geographic origin. Geographically distinct clusters are highlighted in the plot.

The PCA also revealed stronger geographical clustering in *S. glabra* compared to *S. petersenii* (Fig. 2). Nonetheless, some geographically close individuals of *S. petersenii*, e.g. from Greece and Ukraine, formed distinct clusters in PCA space, suggesting their genetic similarity (Fig. 2A). However, individuals of *S. glabra* formed compact and geographically clearly separated groups (individuals from Spain, Greece, Finland, France, and Poland; Fig. 2B), consistent with the STRUCTURE results. The results of PCoA supported these patterns of genetic differentiation in both species (Fig. S3).

### Effects of environmental variables on population structure

**Figure 3:**
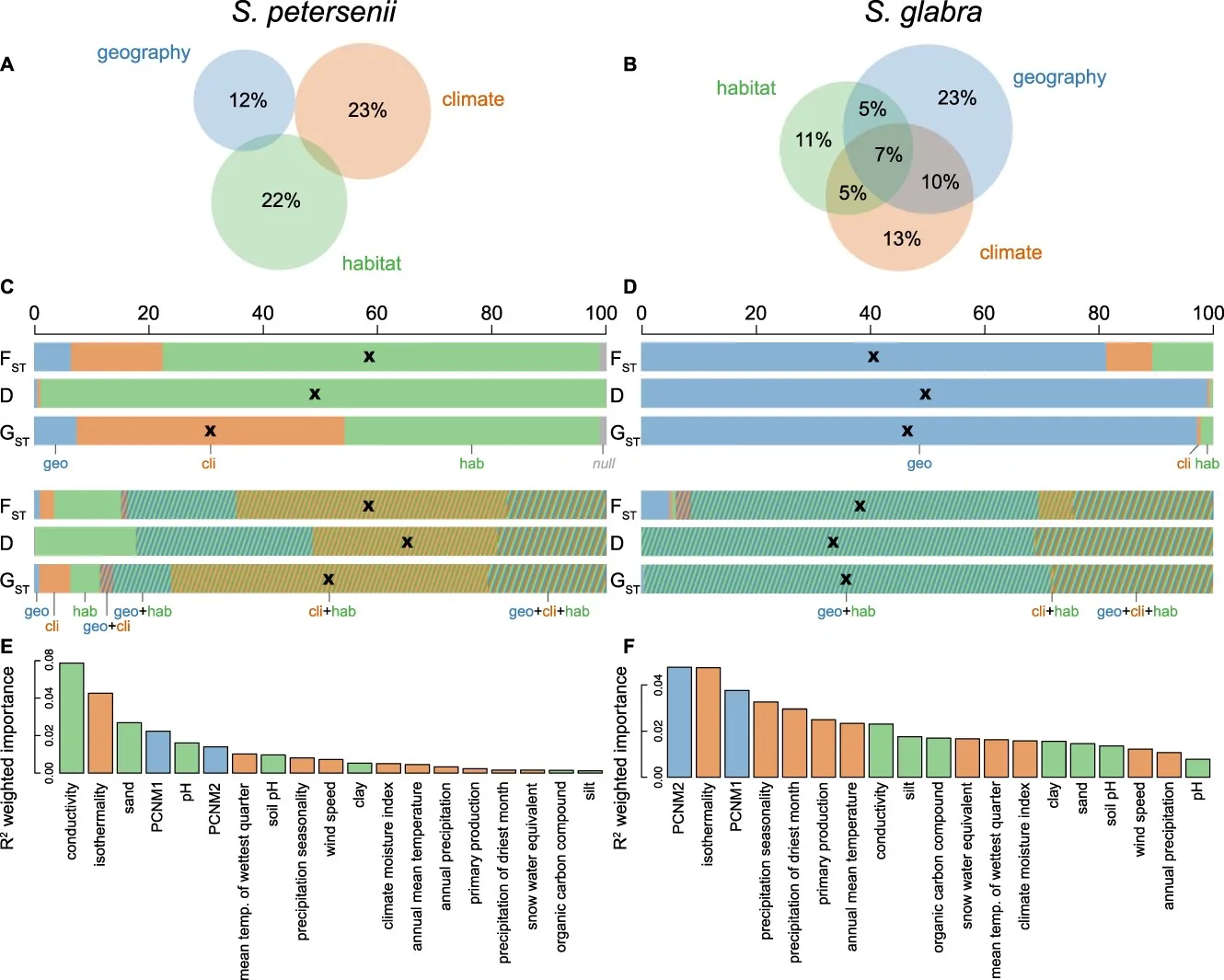
Genotype–environment associations of *S. petersenii* (A, C, E) and *S. glabra* (B, D, F). (A, B) The proportion of loci associated with geographical distance, climate, and habitat. (C, D) Relative contribution of predictors (geo—geography; cli—climate; HAB—habitat), based on weighted deviance information criterion (DIC) coefficients from GLMM analysis, explaining differentiation of populations using *F*_ST_, *D*_Jost_, and *G*_ST_ genetic divergence metrices, and analyzed with (upper plots) and without interaction (lower plots). The best-supported models are indicated by an “X.” (E, F) The importance of climate, habitat, and geography in explaining genetic variation across populations, as determined by the gradient forest analysis.

The relationship between geographic, environmental, and genetic distances was assessed using multiple analytical approaches. First, the relations were visualized with variation partitioning analyses (Fig. 3A and B). All models explained a large proportion of the variation and were highly significant (ANOVA, *P* = .001). For *S. petersenii*, climate and habitat explained comparable proportions of genetic variation (net effects of 23% and 22%), whereas their shared contribution was negligible. By contrast, in *S. glabra*, spatial factors explained the largest proportion of genetic variation (net effect of 23%), while climate and habitat made comparatively smaller independent contributions (net effects of 13% and 11%, respectively). Generalized linear mixed models (GLMMs) incorporating climatic, habitat, and geographical variables (Fig. 3C and D) revealed contrasting patterns between the two species. In *S. petersenii*, habitat variables were the strongest predictors, both independently and in combination with climate and/or geography. In contrast, genetic variation in *S. glabra* was primarily explained by geographical variables, followed by interactions between geography and either habitat or climate.

Gradient forest analyses (GFM) evaluated the relative importance of environmental predictors based on cumulative importance curves, where steeper gradients indicate stronger relative importance of the predictor ([60]; Fig. 3E and F). For *S. petersenii*, the most influential predictors were conductivity, isothermality, sandy soil, and geographic origin. For *S. glabra*, the cumulative importance curves revealed a smoother transition, suggesting a more gradual influence of environmental variables. In addition to geographic distance, isothermality and precipitation emerged as key predictors.

**Figure 4:**
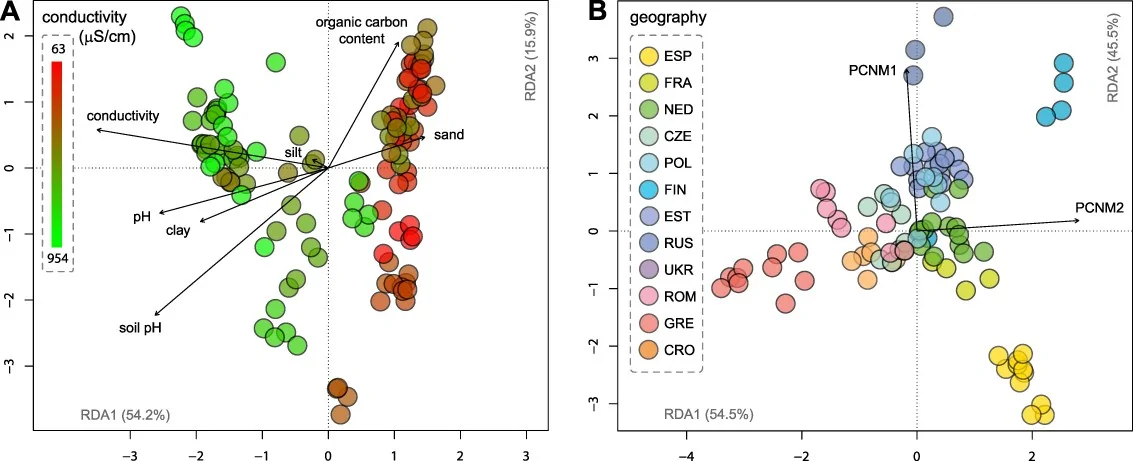
RDA ordination plots showing the associations of genetic diversity with habitat variables for *S. petersenii* (A) and geographical predictors for *S. glabra* (B). The strains are distinguished based on the selected factors characterizing the sampling sites (conductivity and geographical area).

Finally, based on the preceding analyses, RDA ordination plots were used to highlight the most informative variable sets: habitat for *S. petersenii* and geography for *S. glabra* (Fig. 4A and B). For *S. petersenii*, habitat-related variation was primarily explained by conductivity, pH, and soil organic carbon. Conductivity was strongly correlated with the first axis of the RDA plot (Fig. 4A). In contrast, the genetic structure of *S. glabra* was primarily shaped by geography, with clear spatial differentiation (Fig. 4B).

## Discussion

Overall, we detected significant genetic differentiation within the two closely related protist species, *S. petersenii* and *S. glabra*. Although both species exhibited clear genetic structuring across Europe, their spatial patterns differed markedly. Our results demonstrate that seemingly similar protists may follow distinct mechanisms of population differentiation: *S. petersenii* is shaped primarily by isolation by ecology, whereas *S. glabra* follows isolation by distance (Fig. 3). This supports the view that both allopatric speciation and ecological divergence in sympatry are common in protists, each advantageous yet species specific.

### Population structure as a driver of protist biogeography

Population structure links microevolutionary processes to large-scale biogeographic patterns in protists. Even cosmopolitan taxa can exhibit strong genetic structuring, reflecting limited dispersal, local adaptation, and historical demographic processes. Within the genus *Synura*, species range from broadly distributed to geographically restricted distributions [29, 31, 61], often associated with distinct environmental preferences [62].

In *S. glabra*, clustering analyses revealed several genetically differentiated populations with a strong geographical pattern and limited migration among regions (Fig. 1). Similar geographically distinct population clusters have been reported for other protists as well [11, 40, 63]. Some studies indicate that each locality harbors a largely separate gene pool [14, 64, 65], a pattern that can set the stage for eventual allopatric speciation. Together, our results and previous studies demonstrate that allopatric speciation can occur in protists despite their large population sizes and presumed high dispersal potential. These findings challenge the traditional view that extensive dispersal and large effective population sizes necessarily prevent geographic isolation from driving speciation [66, 67].

By contrast, *S. petersenii* shows a more complex pattern. Only one population from Greece is strongly differentiated, while others lack clear geographic clustering and instead reflect habitat-driven divergence. Previous work [8] described three core populations plus transitional admixed groups, with conductivity as the decisive factor. Our study detects finer-scale structure across Europe, again highlighting conductivity as the major driver (Fig. 3). A comparable pattern was reported for the freshwater alga *Gonyostomum* [34], which showed ecological divergence among European population clusters. The distributional pattern of *S. petersenii* also resembles a mosaic of isolated patches without clear geographic boundaries, as reported for other microorganisms [68, 69].

Protist distributions are now widely recognized as constrained by both ecological and spatial factors [4, 6, 70, 71]. Building on this consensus, we next explore the role of dispersal and environmental factors in shaping protist diversification.

### The role of dispersal in protist diversification

Protist ranges appear to be partly shaped by a distance–decay relationship, reflecting limits to their dispersal capacity [72–74]. This dispersal is likely mediated primarily by hydrological connectivity among aquatic systems, wind currents, and transport by macroorganisms [75]. Waterfowl (aquatic birds, including ducks, geese, swans, and other species associated with freshwater habitats) have been proposed as feasible vectors, supported by the detection of viable protist species on their feathers and other body surfaces [76]. The high degree of admixture and the occurrence of genetically similar populations across widely separated regions of Europe suggest that *S. petersenii* undergoes effective long-distance dispersal. Why, then, does the closely related *S. glabra* exhibit a strikingly different pattern of population structure? One possible explanation lies in species-specific differences in the ability to withstand the stresses associated with long-distance dispersal through the formation of resistant stages, including silica-encased cysts or mucilage-coated palmella, which remain colonial but shed flagella. While palmella may adhere more effectively to vectors, stomatocysts are generally more durable and capable of long-term survival [77]. Although palmella formation has been reported for several *Synura* species [78, 79] and cysts are common in sediments [80], their transfer remains poorly understood. We propose that *S. glabra* disperses mainly via a stepping-stone model [81], with gene flow among neighboring localities, whereas *S. petersenii* withstands migratory stress more effectively and disperses across greater distances. This is supported by our field observations of *Synura* palmella stages. Although rare, we documented palmella formation, during which multiple colonies within a population transformed simultaneously. These stages were observed in *S. petersenii* and *S. heteropora*, but never in *S. glabra* (Fig. S4). Still, cysts may represent the primary resistant stage for *S. glabra* and thus serve as alternative dispersal vectors.

Nevertheless, both species are capable of long-distance dispersal. This inference is supported by a remote Central Asian population that exhibited only limited genetic differentiation from European population (Fig. 1). The absence of a distinct Asian cluster is unexpected since large-scale dispersal events are generally considered too infrequent to prevent divergence [12, 34, 68, 82, 83]. Rare but effective dispersal may occur via migratory waterfowl, with cysts surviving gut passage [84]. Anthropogenic pathways such as aquaculture, fish stocking, or boat traffic could also contribute, as demonstrated by human-mediated introductions of diatoms [20, 85].

### Environmental factors driving the diversification

Environmental conditions are consistently demonstrated as key drivers shaping protist traits, ultimately determining population composition and adaptive capacity. Protists respond sensitively to gradients of temperature, salinity, pH, and nutrients, which influence distribution and community assembly [21, 72, 86–88]. Environmental filtering has been shown to structure protist communities across both aquatic and terrestrial ecosystems, with climate and habitat variables often outweighing purely stochastic processes [89, 90].

Among the most decisive habitat variables for both *Synura* species were pH and conductivity (Fig. 4), which broadly characterize local conditions relevant to many chrysophytes [91, 92]. While pH directly influences physiological processes, conductivity may serve as a proxy for trophic status and nutrient availability. Environmental gradients associated with nutrient availability have been shown to drive selection in protists, shaping patterns of genetic differentiation and ultimately influencing community composition [93, 94]. Although these conditions fluctuate, the niche width of both species falls within defined tolerance ranges [92], suggesting underlying deterministic mechanisms. Consistent with Jadrná and Škaloud [28], *S. glabra* prefers higher pH, conductivity, and nutrient levels, whereas *S. petersenii* has a broader niche and lower sensitivity to environmental fluctuations. Their ecological preferences overlap substantially, explaining widespread European distribution.

Species with broad tolerance ranges tend to diversify more frequently in response to environmental conditions than species with narrower ecological limits [95]. Therefore, it was reasonable to expect similar ecological diversification in *S. petersenii* and *S. glabra.* However, our analyses revealed contrasting drivers of population differentiation at the same geographical scale: genetic diversity in *S. petersenii* was shaped predominantly by habitat variables, whereas in *S. glabra* geographic distance was the primary determinant (Fig. 3). The size of the study area matters, as climate and habitat were suggested to drive speciation at local scales, while distance–decay patterns should dominate globally [8, 96]. Our results therefore demonstrate that diversification factors depend strongly on the protist species under investigation, and even closely related taxa may employ fundamentally different strategies of distribution and diversification.

## Conclusion

Our study underscores that generalizations about protist biology are inherently limited. Even within a single genus, closely related species may follow divergent evolutionary trajectories, shaped by distinct balances of dispersal, ecological tolerance, and historical contingency. This variability cautions against treating protists as a uniform group with predictable biogeography or speciation dynamics—e.g. assuming that large population sizes and high dispersal capacities universally prevent geographic isolation, or that ecological diversification proceeds in similar ways across taxa. Instead, species-specific approaches that integrate molecular, ecological, and biogeographical perspectives are needed to capture the true complexity of protist evolution. Recognizing this diversity not only refines our understanding of microbial evolution but also highlights protists as powerful models for exploring how multiple mechanisms interact to generate and sustain global biodiversity.

## Supplementary Material

Web_Material_ycag218

## Data Availability

The genetic data have been deposited in the NCBI SRA database (BioProject ID PRJNA1371619) at https://www.ncbi.nlm.nih.gov/bioproject/PRJNA1371619. The R scripts used to analyze the data are available on Mendeley Data: https://doi.org/10.17632/pngtj6ymnp.3.
