# Supplementary material for: Beyond protist generalizations: different evolutionary trajectories in closely related microbial species
Source: ISME Commun. 2026 Mar 8;6(1):ycag218. doi: 10.1093/ismeco/ycag218 (PMC13494791; doi:10.1093/ismeco/ycag218)
Supplement: Supplementary file 1 [file web_material_ycag218.zip › Supplementary_materials_ycag218_Fig S3.pdf]

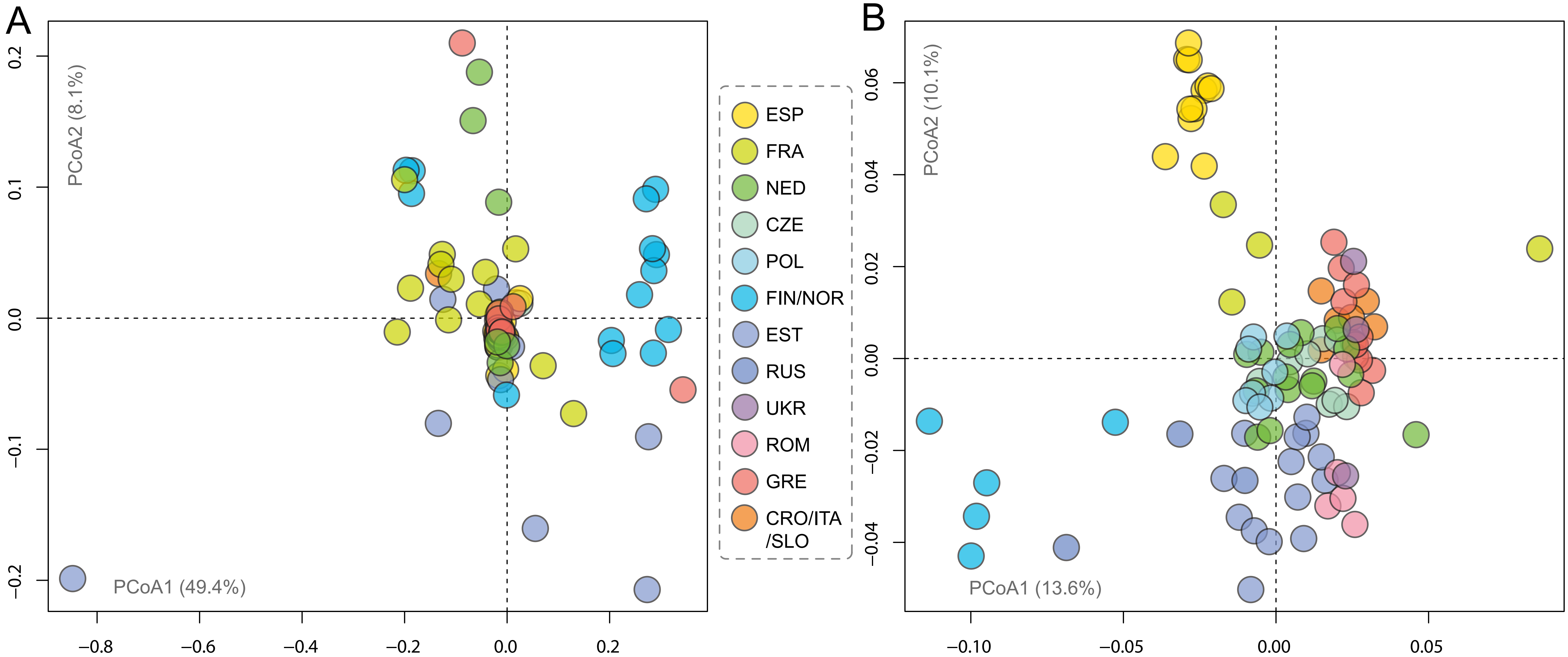
A
B
PCoA1 (49.4%)
PCoA2 (8.1%)
PCoA1 (13.6%)
PCoA2 (10.1%)
ESP
FRA
NED
CZE
POL
FIN/NOR
EST
RUS
UKR
ROM
GRE
CRO/ITA /SLO
−0.8
−0.6
−0.4
−0.2
0.0
0.2
0.1
−0.1
−0.10
−0.05
0.00
0.05
0.06
0.04
0.02
−0.02
−0.04
