# Supplementary material for: Beyond protist generalizations: different evolutionary trajectories in closely related microbial species
Source: ISME Commun. 2026 Mar 8;6(1):ycag218. doi: 10.1093/ismeco/ycag218 (PMC13494791; doi:10.1093/ismeco/ycag218)
Supplement: Supplementary file 1 [file web_material_ycag218.zip › Supplementary_materials_ycag218_Fig S2.pdf]

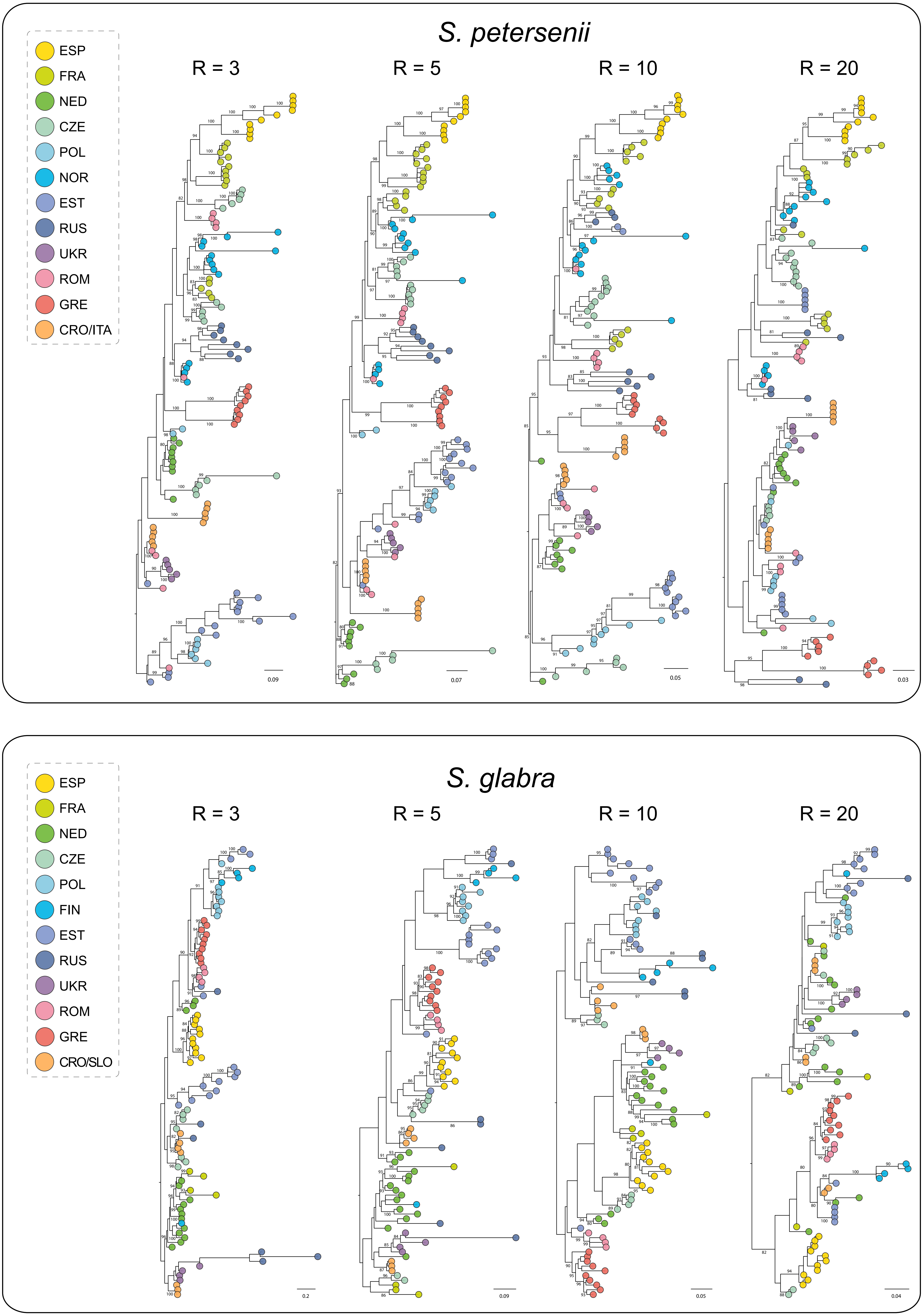

S. petersenii
ESP
FRA
NED
CZE
POL
NOR
EST
RUS
UKR
ROM
GRE
CRO/ITA
R = 3
R = 5
R = 10
R = 20
0.09
0.07
0.05
0.03
S. glabra
ESP
FRA
NED
CZE
POL
FIN
EST
RUS
UKR
ROM
GRE
CRO/SLO
R = 3
R = 5
R = 10
R = 20
0.2
0.09
0.05
0.04
