# Supplementary material for: Beyond protist generalizations: different evolutionary trajectories in closely related microbial species
Source: ISME Commun. 2026 Mar 8;6(1):ycag218. doi: 10.1093/ismeco/ycag218 (PMC13494791; doi:10.1093/ismeco/ycag218)
Supplement: Supplementary file 1 [file web_material_ycag218.zip › Supplementary_materials_ycag218_Fig S1.pdf]

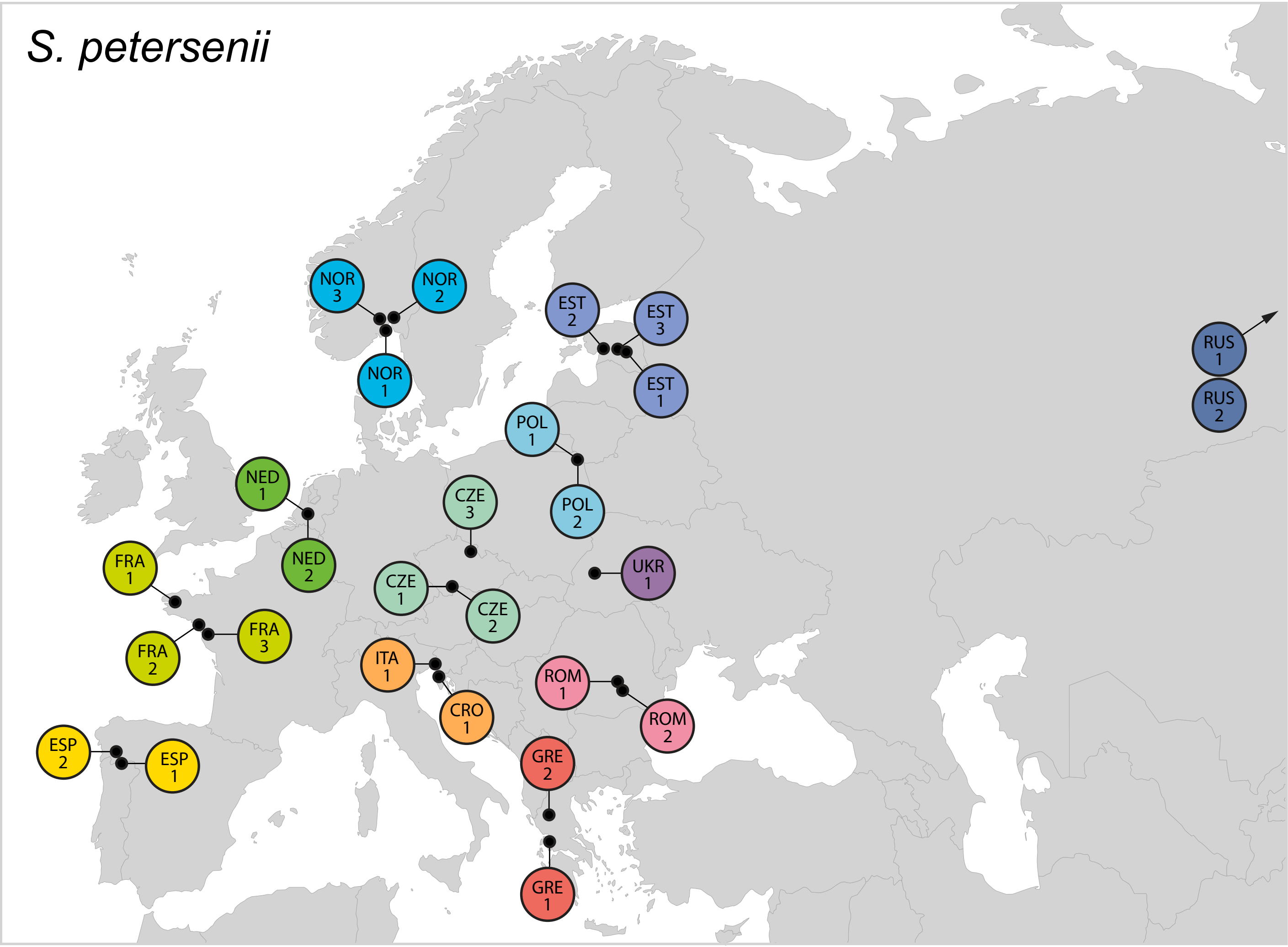
S. petersenii
NOR 3
NOR 2
NOR 1
EST 2
EST 3
EST 1
RUS 1
RUS 2
POL 1
POL 2
NED 1
NED 2
CZE 3
CZE 1
CZE 2
UKR 1
FRA 1
FRA 3
FRA 2
ITA 1
CRO 1
ROM 1
ROM 2
ESP 2
ESP 1
GRE 2
GRE 1

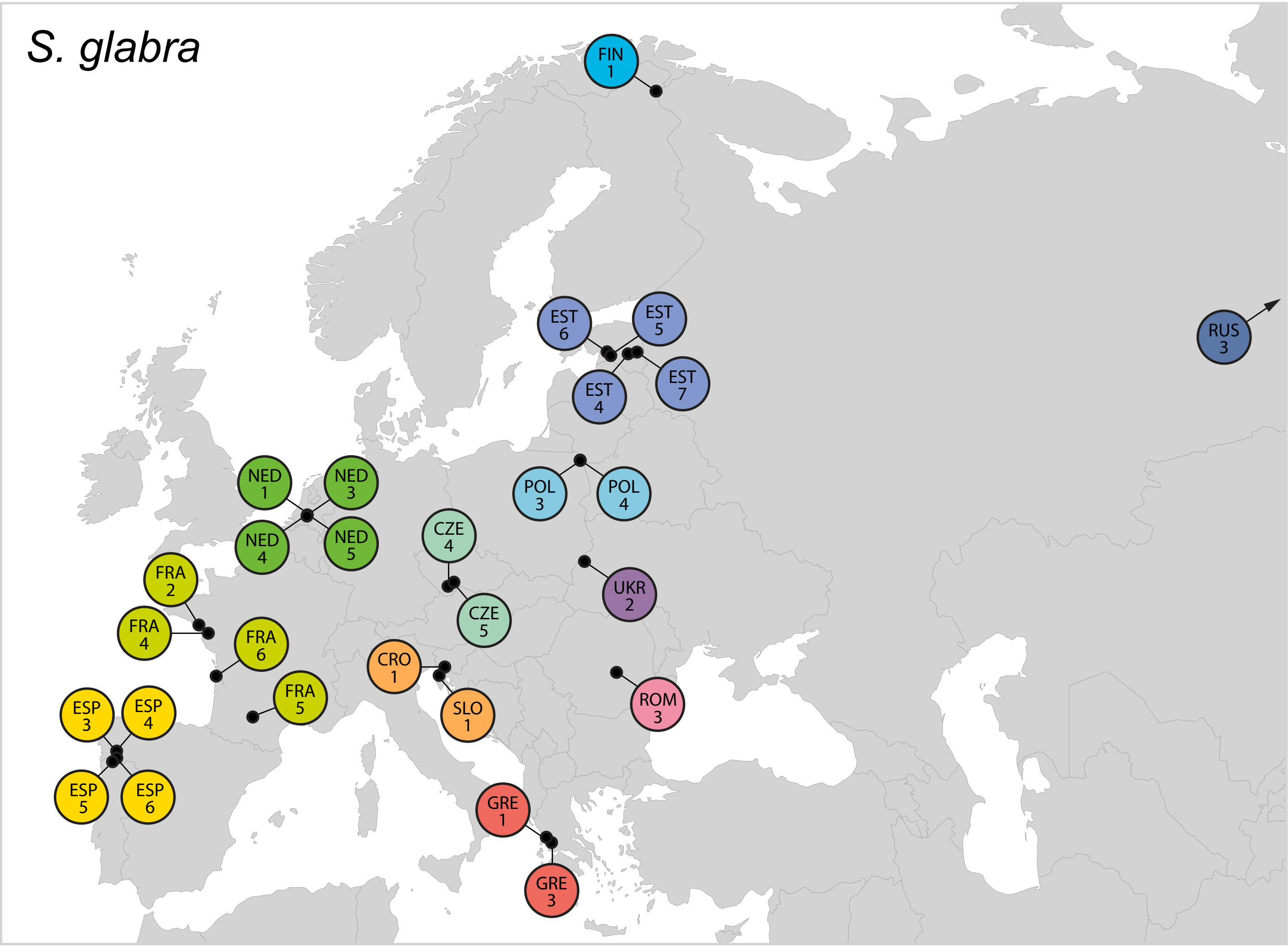
S. glabra
FIN 1
EST 6
EST 5
EST 4
EST 7
RUS 3
NED 1
NED 3
NED 4
NED 5
POL 3
POL 4
CZE 4
CZE 5
UKR 2
FRA 2
FRA 4
FRA 6
FRA 5
CRO 1
SLO 1
ROM 3
ESP 3
ESP 4
ESP 5
ESP 6
GRE 1
GRE 3
