# Supplementary material for: Beyond protist generalizations: different evolutionary trajectories in closely related microbial species
Source: ISME Commun. 2026 Mar 8;6(1):ycag218. doi: 10.1093/ismeco/ycag218 (PMC13494791; doi:10.1093/ismeco/ycag218)
Supplement: Supplementary file 1 [file web_material_ycag218.zip › Supplementary_materials_ycag218.docx]

SUPPORTING INFORMATION for

**Beyond protist generalizations: different evolutionary trajectories in closely related microbial species**

Iva Jadrná^1,2^, Zuzana Škvorová^1^, Pavel Škaloud^1^*

^1^ Charles University, Faculty of Science, Department of Botany, Benátská 2, 128 00 Praha 2, Czech Republic

^2^ Charles University, Faculty of Science, Department of Zoology, Viničná 7, 128 00 Praha 2, Czech Republic

* Corresponding author: Pavel Škaloud, skaloud@natur.cuni.cz

Charles University, Faculty of Science, Department of Botany, Benátská 2, 128 00 Praha 2, Czech Republic

Running title: Different evolutionary trajectories in protist species.

Keywords: biogeography, dispersal, ecology, population differentiation, protists, restriction-site associated DNA (RAD) sequencing, speciation, *Synura*

Table S1: Origin and sampling details of investigated strains, including Bioproject and Biosample numbers.

Table S2: Population genetic metrics of *S. petersenii* and *S. glabra* populations. For the geographic origin of populations, see the Table S1 and Fig. S1. Table content: Pop – population code, Ind – number of individuals in each population, All sites – total number of nucleotide positions across all loci retained after filtering, Var sites – number of variant sites, % polym – percentage of polymorphic sites, Priv. alleles – alleles found only in a given population and absent in all others, π – nucleotide diversity, H_O_ – observed heterozygosity, H_E_ – expected heterozygosity, He, F_IS_ – inbreeding coefficient.

Fig. S1: Overview of the geographic origin of all *S. petersenii* and *S. glabra* samples analysed in this study. Population abbreviations correspond to the sampling localities of individual accessions.

Fig. S2: Results of loci filtering, assessed by maximum likelihood analyses of filtered datasets. For each dataset, four different R thresholds (minimum percentages of individuals across populations required to process a locus) were used.

Fig. S3: PCoA ordination plots showing genetic relationships between the strains of *S. petersenii* (A) and *S. glabra* (B) based on SNPs acquired from sdRAD-seq. The strains are color coded based on their geographic origin.

Fig. S4: Palmella formation in *S. petersenii* (A-D) and *S. heteropora* (E-H). Vegetative colonies (A, E), mature palmella stages (B-C, F-G), and silica scales covering the mucilage. Scale bars: 10 μm (A-C, E-G), 1 μm (D, H).
